# Surface (S) Layer Proteins of *Lactobacillus acidophilus* Block Virus Infection via DC-SIGN Interaction

**DOI:** 10.3389/fmicb.2019.00810

**Published:** 2019-04-16

**Authors:** Mariano Prado Acosta, Eileen M. Geoghegan, Bernd Lepenies, Sandra Ruzal, Margaret Kielian, Maria Guadalupe Martinez

**Affiliations:** ^1^Laboratorio de Bacterias Gram Positivas, Departamento de Química Biológica-IQUIBICEN, Facultad de Ciencias Exactas y Naturales, Universidad de Buenos Aires-CONICET, Buenos Aires, Argentina; ^2^Department of Cell Biology, Albert Einstein College of Medicine, New York, NY, United States; ^3^Immunology Unit and Research Center for Emerging Infections and Zoonosis, University of Veterinary Medicine Hannover, Hanover, Germany

**Keywords:** S-layer, DC-SIGN, alphavirus, flavivirus, *Lactobacillus*

## Abstract

Alphaviruses and flaviviruses are important human pathogens that include Chikungunya virus (CHIKV), Dengue virus (DENV), and Zika virus (ZIKV), which can cause diseases in humans ranging from arthralgia to hemorrhagic fevers and microcephaly. It was previously shown that treatment with surface layer (S-layer) protein, present on the bacterial cell-envelope of *Lactobacillus acidophilus*, is able to inhibit viral and bacterial infections by blocking the pathogen’s interaction with DC-specific intercellular adhesion molecule 3-grabbing non-integrin (DC-SIGN), a trans-membrane protein that is a C-type calcium-dependent lectin. DC-SIGN is known to act as an attachment factor for several viruses including alphaviruses and flaviviruses. In the present study, we used alphaviruses as a model system to dissect the mechanism of S-layer inhibition. We first evaluated the protective effect of S-layer using 3T3 cells, either wild type or stably expressing DC-SIGN, and infecting with the alphaviruses Semliki Forest virus (SFV) and CHIKV and the flaviviruses ZIKV and DENV. DC-SIGN expression significantly enhanced infection by all four viruses. Treatment of the cells with S-layer prior to infection decreased infectivity of all viruses only in cells expressing DC-SIGN. *In vitro* ELISA experiments showed a direct interaction between S-layer and DC-SIGN; however, confocal microscopy and flow cytometry demonstrated that S-layer binding to the cells was independent of DC-SIGN expression. S-layer protein prevented SFV binding and internalization in DC-SIGN-expressing cells but had no effect on virus binding to DC-SIGN-negative cells. Inhibition of virus binding occurred in a time-dependent manner, with a significant reduction of infection requiring at least a 30-min pre-incubation of S-layer with DC-SIGN-expressing cells. These results suggest that S-layer has a different mechanism of action compared to mannan, a common DC-SIGN-binding compound that has an immediate effect in blocking viral infection. This difference could reflect slower kinetics of S-layer binding to the DC-SIGN present at the plasma membrane (PM). Alternatively, the S-layer/DC-SIGN interaction may trigger the activation of signaling pathways that are required for the inhibition of viral infection. Together our results add important information relevant to the potential use of *L. acidophilus* S-layer protein as an antiviral therapy.

## Introduction

Alphaviruses and flaviviruses are medically significant and widely distributed viral pathogens for which vaccines and antiviral therapies are urgently needed. Arthritogenic alphaviruses, such as Chikungunya virus (CHIKV), are mosquito-borne viruses that cause severe polyarthritis and myositis in humans. Flaviviruses, such as the emerging pathogen Zika virus (ZIKV) and dengue virus (DENV), are also transmitted by the same mosquitoes and have been associated with congenital Zika syndrome in fetuses and severe hemorrhagic fever, respectively (Hamel et al., 2015). Beginning in December 2013, the Pan American Health Organization (PAHO) and World Health Organization (WHO) have classified CHIKV, DENV, and ZIKV as emerging public health problems in the Americas. The WHO estimates that DENV is endemic in 100 countries and 50–100 million DENV infections occur yearly^1^. These numbers could be underestimates due to unreported and misclassified cases. To date ZIKV outbreaks and viral transmission have been identified in 86 countries in four continents^2^ while CHIKV outbreaks have been identified in more than 40 countries in the Americas (Morrison, 2014).

During natural transmission, alphaviruses and flaviviruses are introduced into the skin through bites by infected mosquitos. Due to their presence in the anatomical site of initial infection, dermal dendritic cells (DCs) are among the first cells to encounter incoming viruses (Klimstra et al., 2003). DCs specifically express on their surface DC-specific intercellular adhesion molecule 3-grabbing non-integrin (DC-SIGN), a C-type (calcium-dependent) lectin specialized for the capture and presentation of foreign antigens (Soilleux, 2003; Koppel et al., 2005). As a type II transmembrane protein, DC-SIGN contains extracellular, transmembrane, and intracellular domains, thereby allowing the protein to perform important functions in cell adhesion, pathogen recognition, and signaling (Zhang et al., 2014; Mayer et al., 2017). Both alphaviruses and flaviviruses infect cells by binding to plasma membrane (PM) receptors, endocytic uptake, and low pH-triggered fusion in the endosomal compartment (Kuhn, 2013; Pierson, 2013). Previous publications have demonstrated the importance of interaction with DC-SIGN during the alphavirus and flavivirus attachment and internalization steps (Navarro-Sanchez et al., 2003; Barba-spaeth et al., 2005; Froelich et al., 2011; Long et al., 2013). These studies highlight the importance of further characterizing the role of DC-SIGN during virus internalization and its potential as a target for novel antiviral therapy.

Surface layer (S-layer) proteins are found in the outermost cell envelope of numerous members of the Archaea and Bacteria, including many species of the genus *Lactobacillus* comprising major bacterial species found in human intestines (Hynönen and Palva, 2013). S-layer proteins are organized into arrays of a single polypeptide non-covalently bound to the bacterial cell surface. They are considered to function as protective coats, in the maintenance of cell shape, in ion exchange in the cell wall, and in adhesion to biotic and abiotic surfaces. We and others have shown that the interaction between the S-layer of *Lactobacillus acidophilus*, one of the major bacterial species found in human intestines, and DC-SIGN can act as a potent inhibitor of JUNV and H9N2 viral infections (Martínez et al., 2012; Gao et al., 2016; Prado Acosta et al., 2016). Given that *L. acidophilus* and S-layer are both categorized as “generally recognized as safe” (GRAS) (Dunne et al., 2001; Mohamadzadeh et al., 2008), there is interest in further characterizing this novel mechanism of inhibition in order to develop new therapeutics that would target alphaviruses and flaviviruses.

In this work, we assayed for an S-layer protective effect in alphavirus and flavivirus infection of DC-SIGN-expressing cells. The alphavirus Semliki Forest Virus (SFV) was then used as a tool to investigate the antiviral mechanism of S-layer in DC-SIGN-expressing vs. control cells. We describe the unexpected binding of S-layer to cells devoid of DC-SIGN but also confirm that the presence of DC-SIGN was essential for S-layer’s antiviral activity. S-layer protein exerted its antiviral effect with different kinetics than mannan, a known viral inhibitor that also acts on DC-SIGN (Yu et al., 2017). Together our results suggest that inhibition of viral entry by S-layer occurs via a novel S-layer/DC-SIGN interaction.

## Materials and Methods

### Isolation of S-Layer Proteins

S-layer proteins were extracted from overnight cultures of *L. acidophilus* ATCC 4356 cells grown in MRS medium at 37°C by using 6 M LiCl. The protein was extensively dialyzed against distilled water overnight at 4°C and after centrifugation (10,000 × *g* 20 min), it was suspended in sterile H_2_O and stored at 20°C (Beveridge et al., 1997). Purity was evaluated by SDS-PAGE, which showed a single band after Coomassie blue staining.

### Cell Lines and Viruses

Vero cells, 3T3 cells, and 3T3 cells stably expressing human DC-SIGN (3T3 DC-SIGN) were cultured at 37°C in Dulbecco’s modified Eagle’s medium containing 10% fetal bovine serum, 100 U penicillin/ml, and 100 μg streptomycin/ml. 3T3 parental and 3T3 DC-SIGN-expressing cells were a kind gift from Vineet N. Kewal Ramani, HIV Drug Resistance Program, NCI. SFV was a well-characterized plaque-purified isolate (Glomb-Reinmund and Kielian, 1998), CHIKV was the vaccine strain 181/25, obtained from Dr. Robert Tesh (University of Texas Medical Branch at Galveston, Galveston, TX, United States), DENV 2 (DENV-2) was strain 16681, and ZIKV was strain IbH obtained from the NIH BEI program. All alphavirus stocks were obtained by propagation in BHK-21 cells while the flaviviruses ZIKV and DENV were propagated in C6/36 mosquito cells.

### Antibodies and Reagents

A rabbit polyclonal antibody raised against the SFV envelope proteins (Ahn et al., 1999) and cross reacting with the CHIKV envelope proteins was used for immunofluorescence experiments (anti-SFV Ab). Rabbit anti-human DC-SIGN (D7F5C) antibodies were purchased from Cell Signaling Technologies. The rabbit polyclonal antibody against S-layer was produced as previously published (Acosta et al., 2008). Mannan from *Saccharomyces cerevisiae* was obtained from Sigma (M7504). Alexa 568-conjugated phalloidin and Alexa 488-, 561-, or 405-conjugated anti-mouse or anti-rabbit antibodies were obtained from Molecular Probes.

### Production of the CLR-Fc Fusion Protein

The cDNA encoding the extracellular part of DC-SIGN was amplified by polymerase chain reaction (PCR) and was then ligated into the pFuse-hIgG1-Fc (primers: FW-5′-GAATTCGTCCAAGGTCCCCAGCTCCAT-3′; RV-5′-CCATGGACGCAGGAGGGGGGTTTGGGGT-3′). CHO-S cells were transiently transfected with the construct using MAX reagent (InvivoGen). CLR–hFc fusion proteins were purified after 4 days of transfection from the cell supernatant using HiTrap protein G HP columns (GE Healthcare, Piscataway, NJ, United States). To confirm its purity, the fusion protein was analyzed by SDS-PAGE and subsequent Coomassie staining and by Western blot using an anti-human IgG-horseradish peroxidase (HRP) antibody.

### ELISA-Based Binding Studies

A special microplate with half-area wells (Greiner Bio-One GmbH, Frickenhausen, Germany) was coated with 50 μl of 1 μg/ml of S-layer protein ON at RT. Non-adherent protein were washed away, and the plate was blocked with buffer containing 1% BSA (Thermo Fisher Scientific/Invitrogen, Darmstadt, Germany) in 1x PBS for 2 h at RT. After washing the wells, 50 μl containing 200 ng of each DC-SIGN, L-SIGN–hFc fusion protein in lectin-binding buffer (50 mM HEPES, 5 mM MgCl_2_, and 5 mM CaCl_2_) was added and incubated for 1 h at RT. Then, a 1:5.000-diluted HRP-conjugated goat anti-human IgG antibody (Dianova) was added for 1 h at RT. Finally, the substrate solution [*o*-phenylenediamine dihydrochloride substrate tablet (Thermo Fisher Scientific), 24 mM citrate buffer, 0.04% H_2_O_2_, 50 mM phosphate buffer in H_2_O] was added to the samples, and the reaction was stopped with 2.0 M sulfuric acid. Data were collected using a Multiskan Go microplate spectrophotometer (Thermo Fisher Scientific) at a wavelength of 495 nm. When competition assays were performed, different concentration of S-layer were incubated with DC-SIGN-hFC protein and subsequently incubated with a precoated plate with Mannan-at 50 μg/ml.

### Cell Viability Assays

Viability of cell cultures under each treatment condition was determined by the MTT assay (Chacon et al., 1997). Briefly, after 24 h of treatment with concentrations ranging from 50 to 400 μg/ml of S-layer, cell cultures were incubated for 2 h in culture medium containing 0.5 mg ml-1 MTT. After 2 h, the incubation buffer is removed and the blue MTT–formazan product is extracted with acidified isopropyl alcohol (0.04 N HCl). After 30 min extraction at room temperature, the absorbance of the formazan solution is read spectrophotometrically at 570 nm.

### Virus Infection and Inhibition Experiments

Monolayers of 3T3 parental, 3T3 DC-SIGN, or Vero cells were cultured in 24-well plates and treated for 60 min at 37°C with different concentrations of S-layer, mannan at 50 μg/ml, or medium as a control. Cells were then infected in the presence of S-layer or mannan with SFV at multiplicity of infection (MOI) = 10, CHIKV at MOI = 5, DENV-2 at MOI = 10, or ZIKV at MOI = 10. After 3 h at 37°C, cells were washed and incubated with fresh media in the presence of 20 mM NH_4_Cl to prevent secondary infection. Cells were fixed with 4% paraformaldehyde (PFA, Electron Microscopy Science) at 24 h post infection (hpi) for the alphaviruses SFV and CHIKV or 48 hpi for the flaviviruses DENV-2 and ZIKV. Cells were permeabilized with 0.2% Triton X-100 for 5 min at room temperature and then stained with antibodies to detect the viral glycoproteins or with Hoechst 33342 (Invitrogen H3570) to visualize cell nuclei. Images were acquired with a Zeiss inverted epifluorescence microscope, and a total of 30 fields of view were randomly acquired in three independent experiments. The total number of cells was quantitated based on the nuclei staining and infected cells were quantitated by their positive expression of the viral antigen. The percentage of infected cells relative to control untreated cells was determined. Statistical significance was calculated by a two-tailed unpaired Student’s *t*-test, as indicated in the figures.

### Adsorption/Internalization Assay

3T3 wild type cells, 3T3 DC-SIGN-expressing cells, and Vero cells were cultured in MatTek glass-bottom culture dishes (number 1.5; P35G-1.5-14-C; MatTek Corporation). Cells were either untreated or incubated with 200 μg/ml S-layer for 1 h at 37°C. SFV (1 × 10^9^ pfu) in Rmed (RPMI without bicarbonate plus 0.2% BSA and 10 mM HEPES) was bound to the cells for 60 min on ice. The cells were then incubated at 37°C in Rmed for 20 min to permit endocytosis, washed on ice three times with PBS, and fixed with 4% PFA for 20 min at room temperature. Cell surface virus was stained with anti-SFV MAb and goat anti-rabbit Ig Alexa Fluor 488 (Green). To visualize internalized virus, cells were then permeabilized with 0.2% Triton X-100 and intracellular virus was stained with anti-SFV Ab and goat anti-rabbit Alexa Fluor 568 (Red). As a result, virus that remains bound to the cell surface without internalization will be stained in yellow/green color while virus that was internalized and thus no longer on the cell surface will only be colored in red. S-layer was detected by S-layer Ab and goat anti-mouse Ig Alexa Fluor 405 (Blue). The samples were analyzed by confocal microscopy and images were captured using a Zeiss Live DuoScan confocal microscope.

### DC-SIGN Endocytosis Assay

DC-SIGN-expressing 3T3 cells were treated with S-layer (200 μg/ml) or media alone for 60 min at 37°C. Cells were then incubated with a rabbit anti DC-SIGN antibody for 15 min at 4°C then fixed and stained with anti-rabbit secondary antibodies Alexa Fluor 488. Other cells were further incubated at 37°C for 30 min, fixed, permeabilized, and stained with an Alexa Fluor 568 secondary antibody. Cells were imaged by confocal microscopy.

### Flow Cytometry

3T3, 3T3 DC-SIGN, or Vero cells were plated in 10 cm dishes and the next day dissociated from the dish using Accutase (Sigma–Aldrich A6964) treatment at 37°C. Cells were resuspended, counted, and aliquoted into an untreated 96-well U-bottom plate. The cells were washed twice in MEM media with 2% FBS, or for cells to be treated with EGTA, washed twice in calcium-deficient MEM without FBS (since it contains calcium). Cells were resuspended in the relevant MEM ± 10 mM EGTA and incubated at 37°C for 30 min to allow for chelation of calcium from surface proteins. Cells were then washed again in the relevant MEM and resuspended in MEM containing S-layer at the concentrations indicated in the legend. Cells were incubated with S-layer for 60 min at 37°C, washed once in PBS, and fixed for 10 min at room temperature with 4% PFA. Following fixation, cells were washed in FACS wash buffer (PBS with 2% BSA and 20 mM EDTA) and then incubated with primary antibody (rabbit anti-S-layer) at 1:1500 for 45 min. Cells were washed twice in FACS wash buffer, and then resuspended in secondary antibody (Life Technologies A11008 goat anti-rabbit-488) at 1:1000 and incubated for 30 min at room temperature. Cells were washed twice, and fluorescent signal was measured by flow cytometry using a BD LSR II Flow Cytometer. Cytometry data were analyzed using FlowJo software.

## Results

### S-Layer Protein From *L. acidophilus* Blocks Virus Infection in a DC-SIGN-Dependent Manner

**Figure 1:**
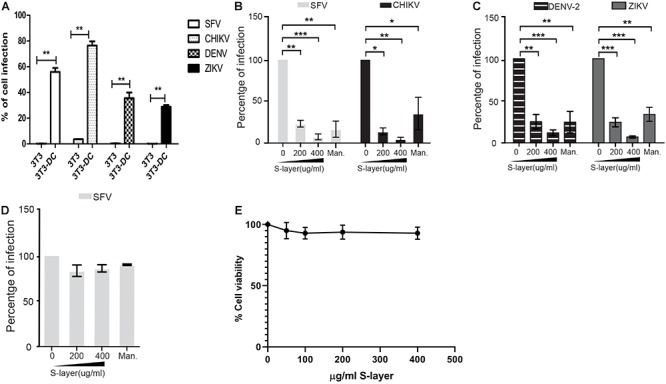
S-layer protein from *L. acidophilus* blocks virus infection in a DC-SIGN-dependent manner. **(A)** Parental 3T3 or 3T3 DC-SIGN cells were infected with the alphaviruses SFV or CHIKV and the flaviviruses DENV-2 or ZIKA. **(B–D)** Cells were preincubated for 60 min at 37°C with the indicated concentrations of S-layer protein or with 50 μg mannan/ml as a control. Cells were then infected with the indicated viruses for 3 h in the continuous presence of S-layer or mannan. **(B)** 3T3 DC-SIGN cells were infected with the alphaviruses SFV or CHIKV. **(C)** 3T3 DC-SIGN cells were infected with the flaviviruses DENV-2 or ZIKA. **(D)** Vero cells were infected with SFV. For the samples in **A–D**, after 3 h infection at 37°C, the cells were transferred to media containing 20 mM NH_4_Cl to prevent secondary infection. Cells were fixed at 24 hpi for alphaviruses **(A,B,D)** or 48 hpi for flaviviruses **(A,C)**, and infection scored by immunofluorescence. Graphs show the percentage of infection normalized to 3T3 DC-SIGN or Vero cells in the absence of treatment. **(E)** Cell viability was determined by MTT assay after a 24 h treatment. Cytotoxicity effect of the protein S-layer protein in 3T3 DC-SIGN cells was studied, and no toxicity was found in the range of S-layer concentrations needed for virus inhibition. All graphs represent the mean and standard deviation of three independent experiments. ^∗^*P* < 0.05, ^∗∗^*P* < 0.01, ^∗∗∗^*P* < 0.001.

We used the well-described 3T3 DC-SIGN model (Wu et al., 2002) to evaluate the effect of S-layer on alphavirus and flavivirus infection. In parental 3T3 cells (which do not express DC-SIGN), infection by the alphaviruses SFV or CHIKV, or the flaviviruses DENV-2 or ZIKV was very low or not detected (Figure 1A). When human DC-SIGN was expressed in these cells, both alphaviruses and flaviviruses efficiently infected the cells (Figure 1A). 3T3 DC-SIGN cells were treated with increasing concentrations of purified S-layer for 60 min prior to and during infection. S-layer inhibited both alphavirus and flavivirus infection in a dose-dependent manner (Figure 1B,C). As predicted, mannan also inhibited infection due to its known block of virus binding to DC-SIGN (Figure 1B,C). To verify that the inhibitory effect of S-layer in SFV infection on 3T3 DC-SIGN cells was due to the interaction between S-layer and DC-SIGN, we also tested Vero cells, which are permissive to viral infection but do not express DC-SIGN (Phanthanawiboon et al., 2014). S-layer treatment of Vero cells produced no effect on SFV infectivity, indicating that the antiviral mechanism of S-layer is DC-SIGN dependent (Figure 1D). Cytotoxicity effect of the protein S-layer protein in 3T3-DC-SIGN cells was determined by MTT assay. As shown in previous studies (Martínez et al., 2012) and as expected by our microscopy observations, no toxicity was caused by the concentrations of S-layer used in this study (IC_50_ = 147.79 μg/ml) (Figure 1E). Taken together, our data indicate that S-layer protein can specifically block DC-SIGN-dependent infection of alphaviruses and flaviviruses.

**Figure 2:**
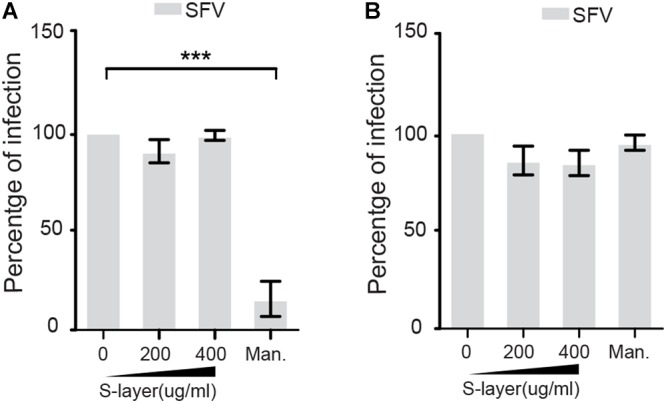
S-layer protein does not directly affect SFV infectivity. SFV was incubated with increasing concentrations of S-layer or mannan for 60 min at 37°C. S-layer protein was then removed from the virus by low speed centrifugation, while mannan remained in the virus mixture after centrifugation. Treated or control virus was then incubated with **(A)** 3T3 DC-SIGN cells or **(B)** Vero cells. After 3 h at 37°C, the virus inoculum was replaced with media containing 20 mM NH_4_Cl to prevent secondary infection. Cells were cultured for a total of 24 hpi and infection scored by immunofluorescence. Graphs show the percentage of infection normalized to the untreated controls, and represent the mean and standard deviation of three independent experiments. ^∗^*P* < 0.05, ^∗∗^*P* < 0.01, ^∗∗∗^*P* < 0.001.

S-layer could potentially cause antiviral effects by inhibiting DC-SIGN-mediated internalization or by acting directly on the viral particle. To address the possibility that S-layer is affecting viral particle stability or fusion activity, we pre-treated SFV for 60 min with increasing concentrations of S-layer, or with mannan as a control. We then removed S-layer protein by taking advantage of its limited solubility in physiologic salt solution (Hynönen and Palva, 2013). S-layer protein was pelleted by low speed centrifugation (10,000 × *g* 20 min) and the treated or control virus was tested for infectivity on 3T3 DC-SIGN cells (Figure 2A) or Vero cells as a non-DC-SIGN-expressing control (Figure 2B). Pretreatment with S-layer did not affect SFV infectivity in the presence or absence of DC-SIGN. In contrast, mannan, a highly soluble compound that is not separated from the virus by low speed centrifugation, blocked virus infection of DC-SIGN-expressing cells while showing no effect on infection of DC-SIGN-negative Vero cells. Thus, there was no direct effect on the SFV viral particle of either S-layer protein or mannan.

### S-Layer Protein Binds Directly but Not Exclusively to DC-SIGN

**Figure 3:**
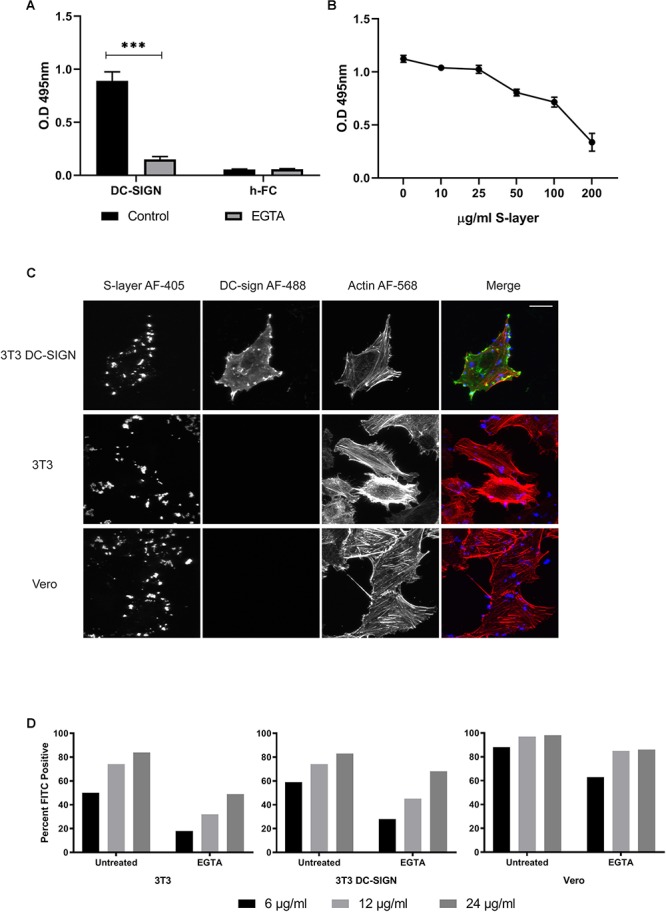
S-layer protein binding to DC-SIGN and to cells. **(A)** ELISA binding assays to S-layer coated plate. The DC-SIGN h-FC fusion protein was diluted in lectin binding buffer or EGTA buffer, incubated with S-layer-coated plates, and washed after incubation to eliminate non-bound protein. Controls were performed using h-FC alone. Unbound proteins were removed by washing and levels of DC-SIGN fusion protein bound to the plate were measured. ^∗∗∗^*P* < 0.001. **(B)** Competition assays using a mannan-coated plate. DC-SIGN-hFc fusion proteins were added to the plate along with increasing concentrations of S-layer. Unbound proteins were removed by washing and levels of DC-SIGN fusion protein bound to the plate were measured. **(C)** 3T3 DC-SIGN, parental 3T3, and Vero cells were incubated with 200 μg/ml of S-layer for 1 h at 37°C. Cells were washed, fixed, and stained with antibodies to detect DC-SIGN (green) and S-layer (blue), and with phalloidin (red) to detect F-actin. Cells were imaged by confocal microscopy. Panel shows confocal extended focus images representative of three independent experiments. Bar = 20 μM. **(D)** Parental 3T3, 3T3 DC-SIGN, and Vero cells were either untreated or treated with 10 mM EGTA, and then incubated 60 min at 37°C with 6, 12, or 24 μg S-layer/ml in the presence or absence of EGTA. Binding was detected by an anti-S-layer antibody and measured using flow cytometry. Data are expressed as percentage of positive cells vs. cells not incubated with S-layer but antibody stained in parallel. The data shown are representative of four independent experiments.

Direct binding of S-layer to DC-SIGN was evaluated by an ELISA assay. This method is based on the expression of a DC-SIGN-hFc fusion protein in which the extracellular part of human DC-SIGN has been fused to the Fc fragment of human IgG1 molecule (Mayer et al., 2018). The S-layer protein was immobilized on the ELISA plate, incubated with the respective DC-SIGN-hFc fusion proteins, and their interaction was quantitated by colorimetric detection (Figure 3A). As expected, a direct interaction was observed by this binding assay (Konstantinov et al., 2008; Lightfoot et al., 2015; Gao et al., 2016). We determined the calcium dependence of the DC-SIGN/S-layer interaction, incubating the proteins in the presence of EGTA. As predicted, the interaction was completely blocked in the presence of EGTA demonstrating its calcium dependency (Figure 3A). Furthermore, we performed competition assays by immobilizing mannan on the ELISA plate and then evaluating the interaction of mannan with DC-SIGN-hFc in the presence of different concentrations of S-layer protein. We observed decreased mannan-DC-sign interaction as the S-layer concentration increased, thus showing a clear competition of mannan and S-layer for binding to DC-SIGN (Figure 3B).

We then evaluated the binding of S-layer to DC-SIGN at the PM by performing confocal microscopy studies. 3T3, 3T3 DC-SIGN, and Vero cells were treated with S-layer protein for 60 min at 37°C, washed extensively, and then immunostained in the absence of permeabilization to detect surface S-layer protein. Phalloidin was used to label actin to delineate cell boundaries, allowing us to cleanly define cell-associated S-layer. As expected, we found that S-layer binds to DC-SIGN-expressing 3T3 cells (Figure 3C). Unexpectedly, S-layer also bound to the surface of the DC-SIGN negative 3T3 and Vero cells. Thus, S-layer binding was observed even in the absence of DC-SIGN, but S-layer only inhibited virus infection in cells expressing DC-SIGN (Figure 1). These results challenge the idea that the S-layer mechanism of action is due to direct blocking of virus absorption to DC-SIGN.

To have a quantitative comparison of the level of S-layer binding across the three cell types used in our experiments, we used flow cytometry to measure S-layer binding to non-permeabilized uninfected Vero, 3T3, and 3T3 DC-SIGN cells. We found that there were not large differences in S-layer binding across the three cell types, despite the lack of DC-SIGN expression in Vero and 3T3 cells (Figure 3D). At 24 μg S-layer/ml, 98% of Vero cells stained positive for S-layer while 83 and 84% of 3T3 and 3T3 DC-SIGN cells, respectively, were positive at this concentration. At a lower concentration of S-layer (6 μg/ml), differences in the percentage of cells bound by S-layer were more pronounced, but the presence of DC-SIGN did not account for the difference in binding since the percentage of Vero cells bound by S-layer was the highest (88%) and 3T3 and 3T3 DC-SIGN cells were approximately equivalent (50 and 59%, respectively). We also analyzed the median fluorescence intensity (MFI) of each cell type as a ratio of the signal obtained with 24 μg S-layer/ml vs. the background staining of no S-layer controls (Supplementary Figure S1). Vero cells demonstrated the highest level of binding (83.7 MFI), followed by 3T3 DC-SIGN (17.5 MFI), and lastly 3T3 cells (12.8 MFI). Together these results indicate that binding of S-layer is not restricted to DC-SIGN and suggest that it can bind to additional surface molecules. To further investigate the role of DC-SIGN in S-layer binding, we tested if calcium chelation with EGTA, a treatment shown to change the structure of DC-SIGN and other C-type lectins (Zhang et al., 2014), would have an effect on S-layer binding. We found that EGTA treatment decreased the percentage of cells bound by S-layer for all cell types but did not eliminate binding (Figure 3D). Treatment with EGTA during binding of 24 μg S-layer/ml decreased the MFI of Vero cells by ∼50%, and the MFI of 3T3 and 3T3 DC-SIGN cells by 82 and 74%, respectively. Together, our results suggest that even though S-layer does binds directly to DC-SIGN, as shown by the ELISA, it is not the only surface molecule mediating S-layer binding.

### S-Layer Decreases Virus Adsorption and Internalization in a DC-SIGN-Dependent Manner

**Figure 4:**
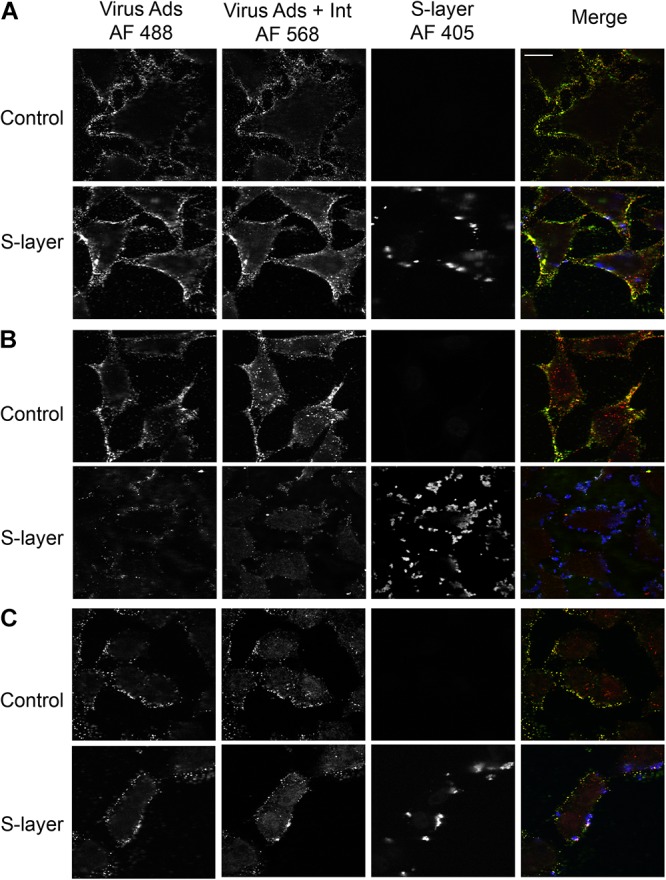
S-layer protein blocks virus internalization in a DC-SIGN-dependent manner. **(A)** Parental 3T3, **(B)** 3T3 DC-SIGN, and **(C)** Vero cells were pretreated for 60 min at 37°C with control media or media containing 200 μg S-layer/ml. Cells were then incubated with SFV for 60 min at 4°C and then shifted to 37°C for 20 min to permit endocytosis, all in the continuous absence or presence of S-layer. Cells were fixed and stained with antibody to the envelope proteins before and after cell permeabilization. These conditions detect virus present at the cell surface in both green/yellow while internalized viruses will only present a red staining. S-layer protein was only detected at the surface and is labeled in blue. Cells were imaged by confocal microscopy. Images from one optical section are shown and are representative of three independent experiments. Magnification of all images is the same, with the bar in the upper right hand panel representing 20 μM.

While we observed binding of the S-layer protein to cells independent of DC-SIGN expression, we only detected a protective effect against infection in cells expressing DC-SIGN. To further understand the mechanism of such protection, we performed assays to dissect the effect of S-layer on virus adsorption and/or internalization. Cells were incubated with SFV for 60 min at 4°C to allow virus adsorption and then incubated 20 min at 37°C to promote virus internalization. Cells were fixed and immunofluorescence staining was performed to detect cell surface adsorbed, but not internalized viruses in the green channel. We then permeabilized the cells and a second immunofluorescence-staining step was performed to detect both adsorbed and internalized virions in the red channel. Note that internalized virus will uniquely stain red in this assay and will be visualized as intracellular puncta, while viruses bound to the PM and not internalized will be stain in a green/yellow (green+red) color. In 3T3 cells that do not express DC-SIGN, S-layer was observed bound to the membrane, as were the viral particles. However, in the absence of DC-SIGN, few viral particles were internalized in 3T3 parental cells either with or without S-layer treatment (Figure 4A). As expected, in 3T3 DC-SIGN cells in the absence of S-layer treatment, viral particles were efficiently adsorbed and internalized (Figure 4B). Both adsorption and internalization were markedly reduced when S-layer treatment was performed. As a control for the effect of S-layer in a permissive cell line that does not express DC-SIGN, we performed similar assays in Vero cells. In agreement with the results in Figure 2, S-layer efficiently bound Vero cells but failed to inhibit viral adsorption and internalization (Figure 4C). These data thus are in keeping with a role for S-layer/DC-SIGN interaction in triggering protection against viral infection.

### S-Layer Protein Does Not Change DC-SIGN Localization or Internalization

**Figure 5:**
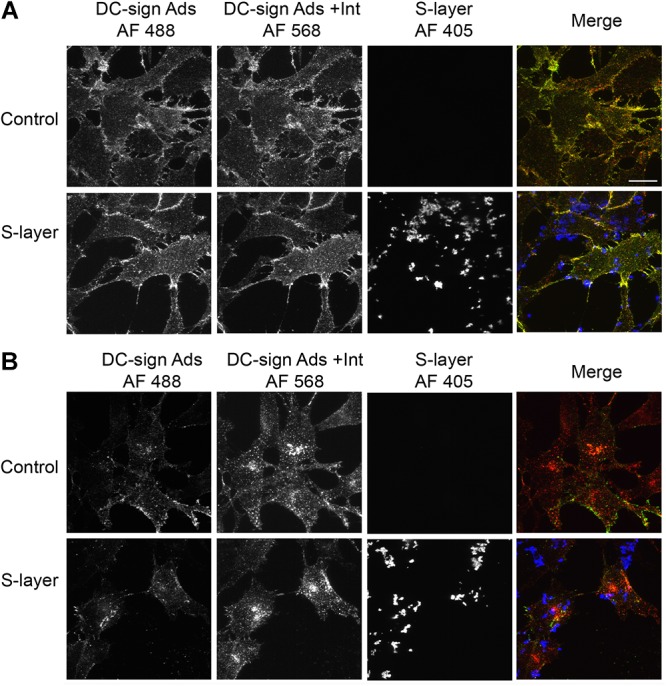
S-layer protein binding does not block DC-SIGN antibody binding or internalization. 3T3 DC-SIGN cells were pretreated with 200 μg S-layer/ml or control media for 60 min at 37°C. Cells were then incubated with anti-DC-SIGN antibody for 15 min at 4°C and **(A)** immediately fixed or **(B)** further incubated for 30 min at 37°C to allow antibody-DC-SIGN complex internalization, and then fixed. Cells were then stained using conditions that detect DC-SIGN antibody on the cell surface (green/yellow) vs. internalized DC-SIGN antibody (red), and with antibodies to detect S-layer (blue). Cells were imaged by confocal microscopy. Images from one optical section are shown and are representative of two independent experiments. Magnification of all images is the same, with the bar in the upper right hand panel representing 20 μM.

Our results suggested that S-layer interaction with DC-SIGN was essential to block virus binding/internalization. A possible explanation could be that S-layer directly or indirectly affects DC-SIGN expression at the PM or its endocytosis/intracellular trafficking. To study the effect of S-layer on DC-SIGN membrane localization and internalization, we performed adsorption/internalization assays using an anti-DC-SIGN antibody. 3T3 DC-SIGN cells were pretreated with control or S-layer-containing-media for 60 min at 37°C. The cells were then either incubated with the anti-DC-SIGN antibody for 15 min at 4°C and fixed (Figure 5A), or further incubated at 37°C to allow internalization of the antibody/DC-SIGN complex (Figure 5B). Figure 5A shows that DC-SIGN was present at the PM of both control and S-layer treated cells. The distribution pattern of DC-SIGN at the PM was unchanged by S-layer treatment. Incubation at 37°C to allow uptake of the DC-SIGN antibody showed that S-layer pretreatment did not block DC-SIGN internalization (Figure 5B). Thus, S-layer did not disrupt DC-SIGN PM localization or its internalization, suggesting that another mechanism is responsible for the antiviral effect of S-layer on DC-SIGN-expressing cells.

### S-Layer Protein Blocks Alphavirus Infection in a Time-Dependent Manner

**Figure 6:**
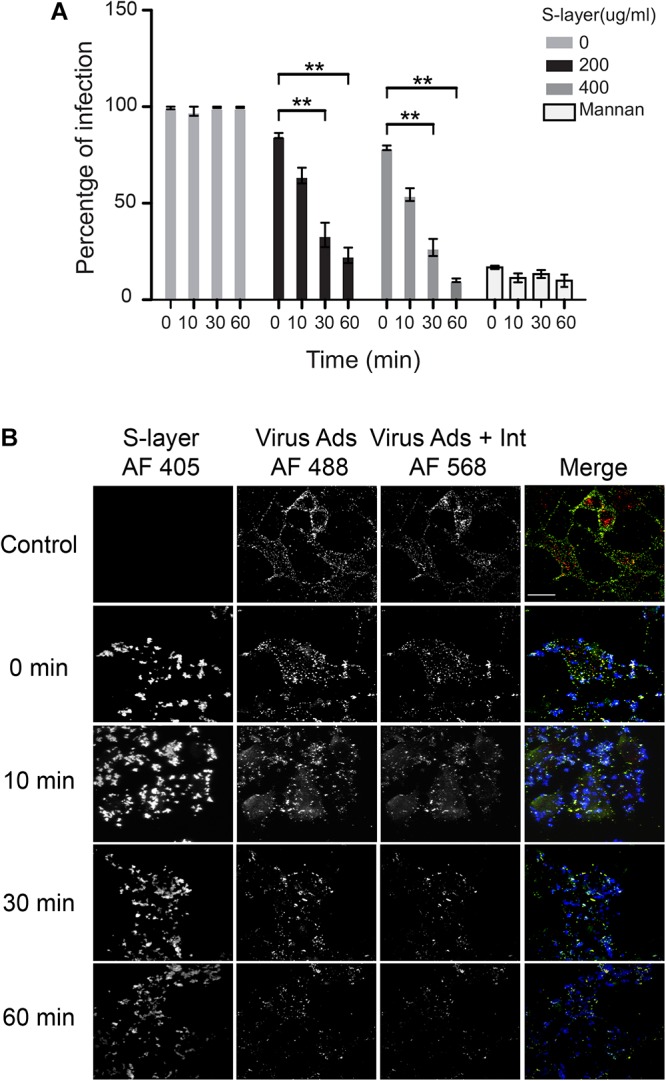
S-layer protein blocks virus binding, internalization and infection in a time-dependent manner. **(A)** 3T3 DC-SIGN cells were pre-incubated at 37°C with the indicated concentrations of S-layer or with mannan (50 μg/ml) for 0, 10, 30, or 60 min, or with control media for 60 min. Cells were then infected with SFV for 3 h at 37°C in the continuous presence S-layer or mannan, where 0 min indicates addition simultaneous with virus. After 3 h at 37°C, media were replaced with media with NH_4_Cl to prevent secondary infection. Cells were cultured for 24 hpi and infection scored by immunofluorescence. Graphs show the percentage of infection normalized to the untreated controls, and represent the mean and standard deviation of three independent experiments. ^∗∗^*P* < 0.01. **(B)** 3T3 DC-SIGN cells were treated at 37°C with 200 μg S-layer/ml for 0, 10, 30, or 60 min, or with control media for 60 min. Cells were then incubated with SFV for 60 min at 4°C and transferred to 37°C for 25 min to permit endocytosis in the continuous presence or absence of S-layer. Cells were fixed and stained with antibody to the envelope proteins before and after cell permeabilization, using conditions that detect virus remaining on the cell surface (green/yellow) vs. internalized virus (red), and with antibodies to detect S-layer (blue). Cells were imaged by confocal microscopy. Images from one optical section/sample are shown and are representative of three independent experiments. Bar = 20 μM.

In an effort to further explore the mechanism of inhibition for S-layer, we decided to compare its antiviral activity to that of the well-characterized DC-SIGN ligand mannan. Mannan is a glycan that is known to directly bind DC-SIGN and block viral particles from engaging the protein, thereby preventing pathogen internalization (Shimojima and Takenouchi, 2014). One potential difference in the mechanism is the kinetics with which each inhibitor operates. Mannan blocks virus infection immediately by sterically preventing virus binding to DC-SIGN, while the time course of the antiviral effects of S-layer was unknown. To determine this, we pretreated cells with S-layer, mannan, or control media for the indicated times at 37°C, then incubated them with SFV for 3 h, and quantitated primary infection (Figure 6A). While inhibition by mannan was observed immediately upon addition (time 0), the protective effect of S-layer was apparent only after 30 or 60 min of pretreatment. Virus adsorption and internalization were also examined by microscopy (Figure 6B). While S-layer association with the cells was observed after all times of addition, S-layer effect on virus adsorption and internalization was stronger after 30 or 60 min of pretreatment. These data suggest that even though S-layer and mannan both act on DC-SIGN at the PM, their mechanisms of action are different.

## Discussion

The incidence of mosquito-borne diseases, including those caused by alphaviruses and flaviviruses, has increased dramatically in recent years. Not only is the range of some mosquitoes expanding to new areas, but some viruses are also adapting to use new species of host mosquitoes (Weaver and Barrett, 2004; Morrison, 2014). Unfortunately, there are currently no available treatments for alphavirus or flavivirus infections, and new therapies or preventative measures are urgently needed. In the present work, we investigated the inhibitory effect of the *L. acidophilus*-derived S-layer protein in alphavirus and flavivirus infection. The goals of this work were to gain new understanding of the inhibitory mechanism of S-layer on viral infection and to potentially identify new antiviral targets in the viral entry process. The well-characterized alphavirus SFV provided an excellent model to dissect the mechanism by which S-layer protein blocks infection.

**Figure 7:**
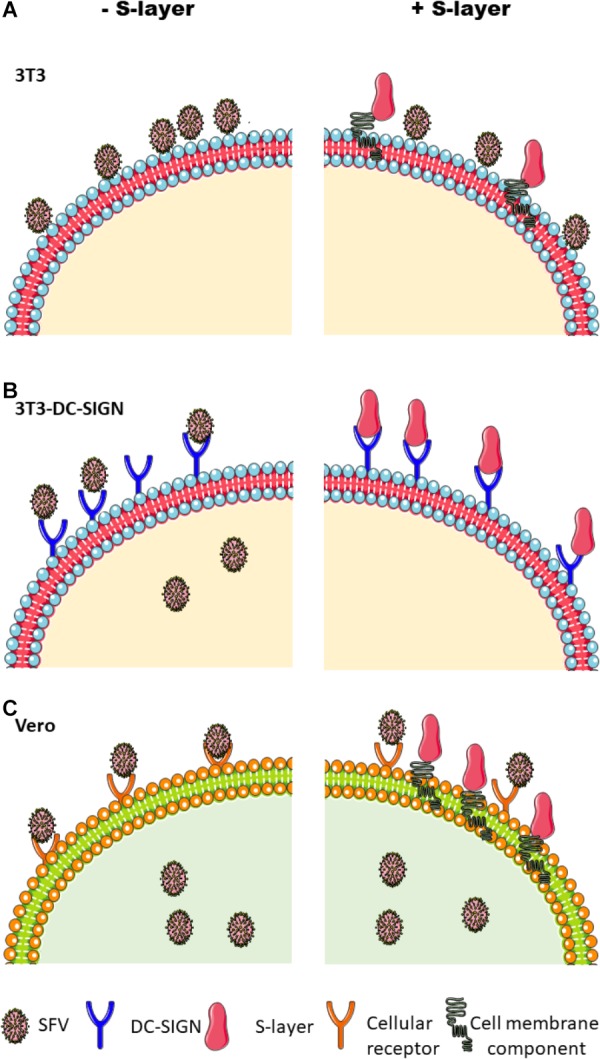
Schematic of the effects of S-layer on alphavirus infection in different cell lines. **(A)** Parental 3T3 cells do not support efficient alphavirus uptake or infection, but virus was detected attached to the plasma membrane. S-layer bound to the cell membrane but did not inhibit virus binding. **(B)** 3T3 DC-SIGN cells are permissive to virus infection, and infection is blocked by S-layer. Treatment with S-layer strongly inhibits virus attachment to the plasma membrane and also inhibits endocytic entry. Unbound viral particles are washed away and thus are not represented in this scheme. **(C)** Vero cells are permissive to infection but do not express DC-SIGN. While Vero cells can bind S-layer, it causes no inhibition of virus binding, endocytic uptake, or infection.

We confirmed that DC-SIGN plays a role in the infection pathway of alphaviruses and flaviviruses by demonstrating that expression of human DC-SIGN on 3T3 cells strongly promoted infection. However, DC-SIGN was not required for infection of all cell types: SFV efficiently infected Vero cells, which do not express DC-SIGN. S-layer treatment of 3T3 DC-SIGN cells prevented infection by SFV, CHIKV, DENV-2, and ZIKV. In contrast, treatment of Vero cells with S-layer had no effect on SFV infection, indicating that the antiviral activity of S-layer requires the presence of DC-SIGN (Figure 7). We ruled out the possibility that S-layer was having a direct irreversible effect on the SFV viral particle by showing that pre-incubation of the virus with S-layer did not affect infectivity. Additionally, we determined that S-layer treatment did not alter DC-SIGN surface expression or inhibit its internalization. Experiments performed in previous publications and in this scoop demonstrated that S-layer does not affect cell viability (Martínez et al., 2012). Therefore, the remaining potential mechanisms likely require an interaction between S-layer and DC-SIGN.

The direct binding between DC-SIGN and S-layer was demonstrated by an ELISA binding assay, and this direct interaction was previously observed by other groups using different methodologies (Konstantinov et al., 2008; Mobili et al., 2009; Taverniti et al., 2013; Lightfoot et al., 2015; Gao et al., 2016; Prado Acosta et al., 2016). Unexpectedly, our immunofluorescence staining and flow cytometry results indicated that S-layer does not require DC-SIGN for binding to cell surface membranes. S-layer bound equally well to 3T3 and 3T3 DC-SIGN cells, and showed the highest binding to DC-SIGN-negative Vero cells. Flow cytometry results also showed that EGTA treatment did not completely eliminate S-layer binding to any of the three cell types; however, EGTA did completely block S-layer biding to DC-SIGN in the ELISA binding assay. Thus, while S-layer can directly interact with DC-SIGN, this does not preclude its binding to other cell surface molecules. This is in keeping with previous evidence that S-layer is capable of binding various cell surface and extracellular matrix proteins, including fibronectin (Hymes et al., 2016).

We performed microscopy experiments to evaluate virus binding and entry in the presence or absence of S-layer. S-layer did not prevent virus binding or internalization in 3T3 parental cells or Vero cells, both of which were devoid of DC-SIGN. While the 3T3 parental cells showed significant binding and little endocytosis of virus, this pattern was not detectably changed by S-layer treatment. In contrast, the 3T3 DC-SIGN cells efficiently bound and internalized SFV, and treatment with S-layer reduced both virus adsorption and internalization. The inhibition of virus-cell binding suggests that the antiviral effect of S-layer could be at least in part due to direct competition with virus particles for binding to DC-SIGN (Figure 7). The reduction in viral entry suggests the possibility that S-layer has additional antiviral mechanism(s).

Mannan is a well-characterized DC-SIGN ligand and a known inhibitor of virus-DC-SIGN binding (Yu et al., 2017). We compared the time course of inhibition by mannan and S-layer in 3T3 DC-SIGN cells. The effect of mannan on viral infection was immediate, with full inhibition even when mannan was added at the same time as the virus. In contrast, significant inhibition by S-layer was only seen after 30 min of pretreatment. There are several potential explanations for the difference in the kinetics of these two DC-SIGN interacting molecules. One could be that S-layer simply requires a longer time for its inhibitory mode of binding to DC-SIGN. Such binding kinetics may not be detected in our immunofluorescence assays. Another explanation is that the DC-SIGN-S-layer interaction is triggering signaling pathways that require time to achieve their antiviral effect. In fact, there is ample evidence in the literature that bacterially derived surface proteins, including S-layer, are capable of triggering a variety of signaling pathways (Tytgat et al., 2016). S-layer can induce production of the anti-inflammatory cytokine IL-10 in immature DCs (Konstantinov et al., 2008), while in macrophage cells, it induces production of TNF-alpha, a pro-inflammatory cytokine (Taverniti et al., 2013). S-layer is also known to be a ligand for TLR2 (Konstantinov et al., 2008; Taverniti et al., 2013), and 3T3 cells are reported to express TLR2 (Lin et al., 2000). Additionally, DC-SIGN is also a mediator of signaling following the recognition of pathogen-associated motifs (Geijtenbeek et al., 2009). Thus, it is possible that S-layer binding to DC-SIGN triggers a time-dependent signaling pathway that inhibits viral entry and infection. Further studies will be necessary to define the mechanisms involved in S-layer inhibition.

Given the evidence that S-layer prevents viral entry, potentially through multiple mechanisms, S-layer proteins can be considered for further investigation as a novel class of antivirals. Recently *L. acidophilus* surface layer protein has been proposed as a treatment for intestinal disorders in humans (Lightfoot et al., 2015). Additionally, Gao et al. (2016) showed that S-layer competes with avian influenza virus (H9N2) for binding to DC-SIGN and inhibits virus infection of DCs. Thus, in addition to the effect of S-layer on alphavirus and flavivirus infection, there may be broader therapeutic possibilities for the use of S-layer protein.

## Author Contributions

MPA, MM, and MK contributed in the conception and design of the study. MPA and SR produced the S-layer protein. MPA carried out the bacterial culture. MPA and MM performed the virus and infection experiments. MM performed the microscopy. EG performed the flow cytometry and analysis. MPA, MM, EG, and MK wrote the manuscript draft. All authors contributed to manuscript revision, and read and approved the submitted version. MPA and BL performed the ELISA binding assay, expression of DC-SIGN-h-Fc fusion proteins, and competition assays.

## Disclaimer

The content of this paper is solely the responsibility of the authors and does not necessarily represent the official views of the National Institute of General Medicine, the National Institute of Allergy and Infectious Diseases, the National Institutes of Health, or the University of Veterinary Medicine Hannover.

## Conflict of Interest Statement

The authors declare that the research was conducted in the absence of any commercial or financial relationships that could be construed as a potential conflict of interest.

## Footnotes

1 https://www.cdc.gov/dengue/epidemiology/index.html

2 https://www.who.int/en/news-room/fact-sheets/detail/zika-virus

## References

[B1] Acosta M. P. Palomino M. M. Allievi M. C. Rivas C. S. Ruzal S. M. (2008). Murein hydrolase activity in the surface layer of *Lactobacillus acidophilus* ATCC 4356. *Appl. Environ. Microbiol.* 74 7824–7827. 10.1128/AEM.01712-08 18931300 PMC2607185

[B2] Ahn A. Klimjack M. R. Chatterjee P. K. Kielian M. (1999). An epitope of the semliki forest virus fusion protein exposed during virus-membrane fusion. *J. Virol.* 73 10029–10039. 10559317 10.1128/jvi.73.12.10029-10039.1999 PMC113054

[B3] Barba-spaeth G. Longman R. S. Albert M. L. Rice C. M. (2005). Live attenuated yellow fever 17D infects human DCs and allows for presentation of endogenous and recombinant T cell epitopes. *J. Exp. Med.* 202 1–6. 10.1084/jem.20051352 16260489 PMC2213233

[B4] Beveridge T. J. Pouwels P. H. Sara M. Kotiranta A. Lounatmaa K. Kari K. (1997). Functions of S-layers. *FEMS Microbiol. Rev.* 20 99–149. 10.1016/S0168-6445(97)00043-0 9276929

[B5] Chacon E. Acosta D. Lemaster J. J. (1997). “9 - Primary Cultures of Cardiac Myocytes as In Vitro Models for Pharmacological and Toxicological Assessments,” in *In Vitro Methods in Pharmaceutical Research*, eds Castell J. V. Jos M.é Gómez-Lechón (San Diego: Academic Press), 209–223. 10.1016/b978-012163390-5.50010-7

[B6] Dunne C. O’Mahony L. Murphy L. Thornton G. Morrissey D. O’Halloran S. (2001). In vitro selection criteria for probiotic bacteria of human origin: correlation with in vivo findings. *Am. J. Clin. Nutr.* 73 386S–392S. 10.1093/ajcn/73.2.386s 11157346

[B7] Froelich S. Tai A. Kennedy K. Zubair A. Wang P. (2011). Virus-receptor mediated transduction of dendritic cells by lentiviruses enveloped with glycoproteins derived from semliki forest virus. *PLoS One* 6:e21491. 10.1371/journal.pone.0021491 21738680 PMC3124512

[B8] Gao X. Huang L. Zhu L. Mou C. Hou Q. Yu Q. (2016). Inhibition of H9N2 virus invasion into dendritic cells by the S-Layer protein from L. acidophilus ATCC 4356. *Front. Cell Infect. Microbiol.* 6:137. 10.3389/fcimb.2016.00137 27826541 PMC5078685

[B9] Geijtenbeek T. B. H. den Dunnen J. Gringhuis S. I. (2009). Pathogen recognition by DC-SIGN shapes adaptive immunity. *Future Microbiol.* 4 879–890. 10.2217/fmb.09.51 19722841

[B10] Glomb-Reinmund S. Kielian M. (1998). fus-1, a pH shift mutant of Semliki Forest virus, acts by altering spike subunit interactions via a mutation in the E2 subunit. *J. Virol.* 72 4281–4287. 9557718 10.1128/jvi.72.5.4281-4287.1998 PMC109658

[B11] Hamel R. Dejarnac O. Wichit S. Ekchariyawat P. Neyret A. Luplertlop N. (2015). Biology of zika virus infection in human skin cells. *J. Virol.* 89 8880–8896. 10.1128/JVI.00354-15 26085147 PMC4524089

[B12] Hymes J. P. Johnson B. R. Barrangou R. Klaenhammer T. R. (2016). Functional analysis of an S-layer-associated fibronectin-binding protein in *Lactobacillus acidophilus* NCFM. *Appl. Environ. Microbiol.* 82 2676–2685. 10.1128/AEM.00024-16 26921419 PMC4836419

[B13] Hynönen U. Palva A. (2013). Lactobacillus surface layer proteins: structure, function and applications. *Appl. Microbiol. Biotechnol.* 97 5225–5243. 10.1007/s00253-013-4962-2 23677442 PMC3666127

[B14] Klimstra W. B. Nangle E. M. Smith M. S. Yurochko A. D. Ryman K. D. (2003). DC-SIGN and L-SIGN Can Act as attachment receptors for alphaviruses and distinguish between mosquito cell- and mammalian cell-derived viruses. *J. Virol.* 77 12022–12032. 10.1128/JVI.77.22.12022 14581539 PMC254289

[B15] Konstantinov S. R. Smidt H. Vos W. M. De Bruijns S. C. M. Kaur S. Valence F. (2008). S layer protein A of *Lactobacillus acidophilus* NCFM regulates immature dendritic cell and T cell functions. *Proc. Natl. Acad. Sci. U.S.A.* 105 19474–19479. 10.1073/pnas.0810305105 19047644 PMC2592362

[B16] Koppel E. A. van Gisbergen K. P. J. M. Geijtenbeek T. B. H. van Kooyk Y. (2005). Distinct functions of DC-SIGN and its homologues L-SIGN (DC-SIGNR) and mSIGNR1 in pathogen recognition and immune regulation. *Cell. Microbiol.* 7 157–165. 10.1111/j.1462-5822.2004.00480.x 15659060

[B17] Kuhn R. J. (2013). “Togaviridae,” in *Fields Virology*, eds Knipe D. M. Howley P. M. (Philadelphia: Lippincott), 629–650.

[B18] Lightfoot Y. L. Selle K. Yang T. Goh Y. J. Sahay B. Zadeh M. (2015). SIGNR 3 -dependent immune regulation by *Lactobacillus acidophilus* surface layer protein A in colitis. *EMBO J.* 34 881–895. 10.15252/embj.201490296 25666591 PMC4388597

[B19] Lin Y. Lee H. Berg A. H. Lisanti M. P. Shapiro L. Scherer P. E. (2000). The lipopolysaccharide-activated toll-like receptor (TLR)-4 induces synthesis of the closely related receptor TLR-2 in adipocytes. *J. Biol. Chem.* 275 24255–24263. 10.1074/jbc.M002137200 10823826

[B20] Long K. M. Whitmore A. C. Ferris M. T. Sempowski G. D. Mcgee C. Trollinger B. (2013). Dendritic cell immunoreceptor regulates chikungunya virus pathogenesis in mice. *J. Virol.* 87 5697–5706. 10.1128/JVI.01611-12 23487448 PMC3648201

[B21] Martínez M. G. Prado Acosta M. Candurra N. A. Ruzal S. M. (2012). S-layer proteins of Lactobacillus acidophilus inhibits JUNV infection. *Biochem. Biophys. Res. Commun.* 422 590–595. 10.1016/j.bbrc.2012.05.031 22595457 PMC7124250

[B22] Mayer S. Moeller R. Monteiro J. T. Ellrott K. Josenhans C. Lepenies B. (2018). C-Type lectin receptor (CLR)-Fc fusion proteins as tools to screen for novel CLR/bacteria interactions: an exemplary study on preselected campylobacter jejuni isolates. *Front. Immunol.* 9:213. 10.3389/fimmu.2018.00213 29487596 PMC5816833

[B23] Mayer S. Raulf M.-K. Lepenies B. (2017). C-type lectins: their network and roles in pathogen recognition and immunity. *Histochem. Cell Biol.* 147 223–237. 10.1007/s00418-016-1523-7 27999992

[B24] Mobili P. de los Ángeles Serradell M. Trejo S. A. Avilés Puigvert F. X. Abraham A. G. De Antoni G. L. (2009). Heterogeneity of S-layer proteins from aggregating and non-aggregating *Lactobacillus kefir* strains. *Int. J. Gen. Mol. Microbiol.* 95 363–372. 10.1007/s10482-009-9322-y 19306111

[B25] Mohamadzadeh M. Duong T. Hoover T. Klaenhammer T. R. (2008). Targeting mucosal dendritic cells with microbial antigens from probiotic lactic acid bacteria. *Expert Rev. Vaccines* 7 163–174. 10.1586/14760584.7.2.163 18324887

[B26] Morrison T. E. (2014). Reemergence of chikungunya virus. *J. Virol.* 88 11644–11647. 10.1128/JVI.01432-14 25078691 PMC4178719

[B27] Navarro-Sanchez E. Altmeyer R. Amara A. Schwartz O. Fieschi F. Virelizier J. L. (2003). Dendritic-cell-specific ICAM3-grabbing non-integrin is essential for the productive infection of human dendritic cells by mosquito-cell-derived dengue viruses. *EMBO Rep.* 4 723–728. 10.1038/sj.embor.embor866 12783086 PMC1326316

[B28] Phanthanawiboon S. A-nuegoonpipat A. Panngarm N. Limkittikul K. Ikuta K. Anantapreecha S. (2014). Isolation and propagation of dengue virus in vero and BHK-21 cells expressing human DC-SIGN stably. *J. Virol. Methods* 209 55–61. 10.1016/j.jviromet.2014.08.023 25205264

[B29] Pierson T. C. (2013). “Flaviviruses,” in *Fields Virology*, eds Knipe D. M. Howley P. M. (Philadelphia: Lippincott), 747–794.

[B30] Prado Acosta M. Ruzal S. M. Cordo S. M. (2016). S-layer proteins from *Lactobacillus* sp. inhibit bacterial infection by blockage of DC-SIGN cell receptor. *Int. J. Biol. Macromol.* 92 998–1005. 10.1016/j.ijbiomac.2016.07.096 27498415

[B31] Shimojima M. Takenouchi A. (2014). Distinct usage of three C-type lectins by Japanese encephalitis virus?: DC-SIGN, DC-SIGNR, and LSECtin. *Arch. Virol.* 159 2023–2031. 10.1007/s00705-014-2042-2 24623090 PMC7087284

[B32] Soilleux E. J. (2003). DC-SIGN (dendritic cell-specific ICAM-grabbing non-integrin) and DC-SIGN-related (DC-SIGNR): friend or foe? *Clin. Sci.* 104 437–446. 10.1042/CS20020092 12653690

[B33] Taverniti V. Stuknyte M. Minuzzo M. Arioli S. De Noni I. Scabiosi C. (2013). S-layer protein mediates the stimulatory effect of *Lactobacillus helveticus* MIMLh5 on innate immunity. *Appl. Environ. Microbiol.* 79 1221–1231. 10.1128/AEM.03056-12 23220964 PMC3568609

[B34] Tytgat H. L. P. Schoofs G. Vanderleyden J. Van Damme E. J. M. Wattiez R. Lebeer S. (2016). Systematic exploration of the glycoproteome of the beneficial gut isolate *Lactobacillus rhamnosus* GG. *J. Mol. Microbiol. Biotechnol.* 26 345–358. 10.1159/000447091 27463506

[B35] Weaver S. C. Barrett A. D. T. (2004). Transmission cycles, host range, evolution and emergence of arboviral disease. *Nat. Rev. Microbiol.* 2 789–801. 10.1038/nrmicro1006 15378043 PMC7097645

[B36] Wu L. Martin T. D. Vazeux R. Unutmaz D. KewalRamani V. N. (2002). Functional evaluation of DC-SIGN monoclonal antibodies reveals DC-SIGN interactions with ICAM-3 do not promote human immunodeficiency virus type 1 transmission. *J. Virol.* 76 5905–5914. 10.1128/jvi.76.12.5905-5914.2002 12021323 PMC136240

[B37] Yu L. Shang S. Tao R. Wang C. Zhang L. Peng H. (2017). High doses of recombinant mannan-binding lectin inhibit the binding of influenza A(H1N1)pdm09 virus with cells expressing DC-SIGN. *APMIS* 125 655–664. 10.1111/apm.12695 28493491

[B38] Zhang F. Ren S. Zuo Y. (2014). DC-SIGN, DC-SIGNR and LSECtin: C-Type Lectins for Infection. *Int. Rev. Immunol.* 33 54–66. 10.3109/08830185.2013.834897 24156700

