# Supplementary material for: Surface (S) Layer Proteins of Lactobacillus acidophilus Block Virus Infection via DC-SIGN Interaction
Source: Front Microbiol. 2019 Apr 16;10:810. doi: 10.3389/fmicb.2019.00810 (PMC6477042; doi:10.3389/fmicb.2019.00810)
Supplement: Supplementary file 1 [file Table_1.DOCX]

**Figure S1. Flow cytometry.** Cells were either untreated or treated with 10mM EGTA, and then incubated 60 minutes at 37°C with 24 µg S-layer/ml. Binding was detected by an anti-S-layer antibody and measured using flow cytometry. The median fluorescence intensity (MFI) of S-layer signal binding to each cell type is expressed as a ratio of the signal obtained with 24 µg S-layer/ml vs the background staining of no S-layer controls. The data shown are an average of three independent experiments, and error bars are standard deviation.
