# Supplementary figures and images for: Surface (S) Layer Proteins of Lactobacillus acidophilus Block Virus Infection via DC-SIGN Interaction
Source: Front Microbiol. 2019 Apr 16;10:810. doi: 10.3389/fmicb.2019.00810 (PMC6477042; doi:10.3389/fmicb.2019.00810)

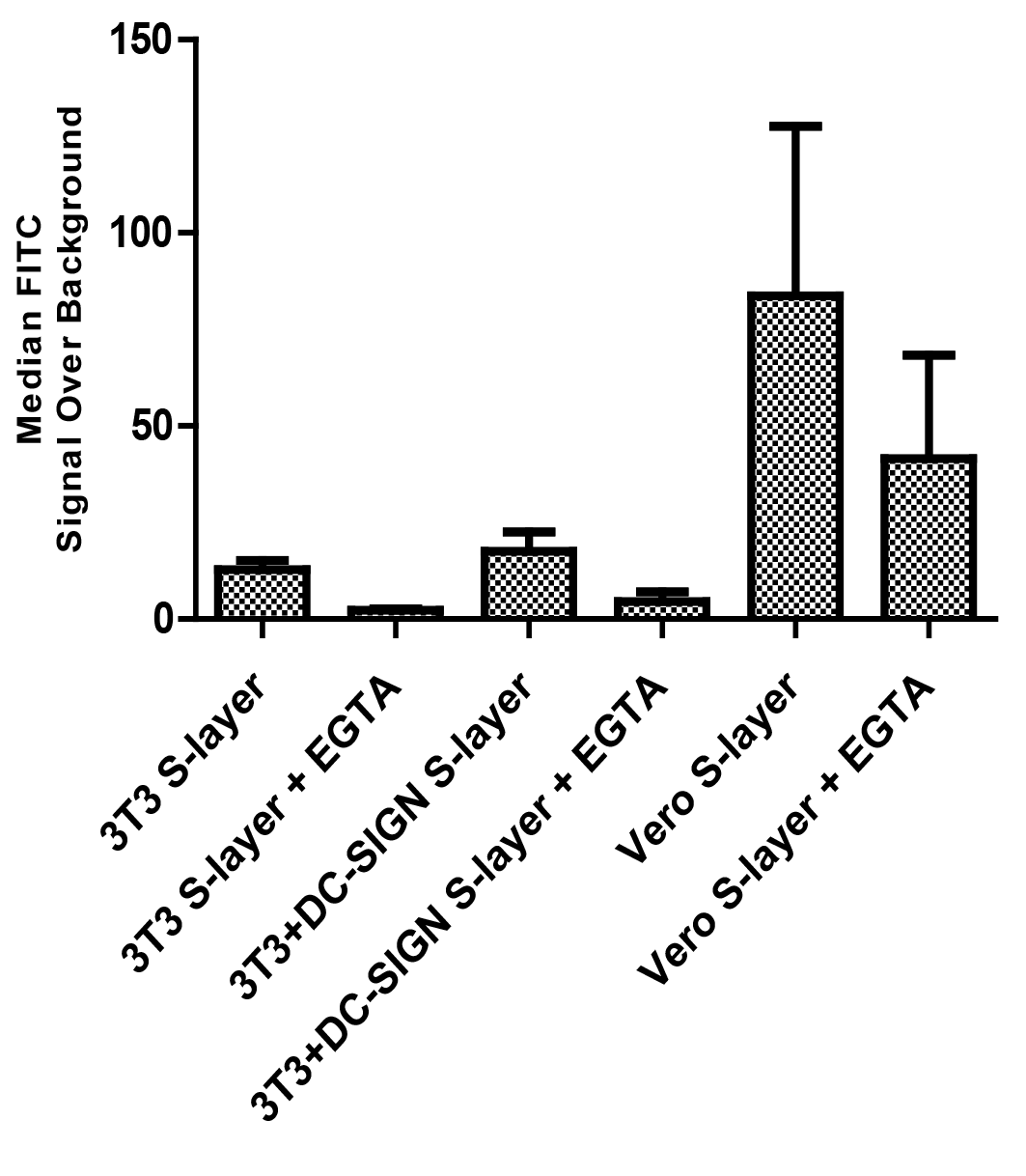

Supplement: Supplementary file 2 [file Image_1.TIF]
